# Mortality of Surgical Operations in the Upper Lake States, Compared with That of Other Regions

**Published:** 1876-08

**Authors:** Edmund Andrews

**Affiliations:** Professor of Princples and Practice of Surgery in Chicago Medical College


### THE MORTALITY OF SURGICAL OPERATIONS IN THE UPPER LAKE STATES, COMPARED WITH THAT OF OTHER REGIONS.

By EDMUND ANDREWS, A.M., M.D.,
Professor of Princples and Practice of Surgery in Chicago Medical College,

Assisted by Thos. B. Lacey, M.D.,
Assistant Surgeon in the National Soldiers' Home.

(Continued.)

### OPINIONS OF AUTHORS AND CONCLUSIONS.

The great authorities on surgery, give us almost no advice about the particular indications for amputation of the arm, but fall back on the established general principles, which are these:

1. The superior extremity is of more value than the inferior, and should be sacrificed with more reluctance.

2. Gangrene and diseases of its joints are less dangerous than in the inferior extremity; hence in certain cases it is less perilous to delay the operation, until its necessity is fully proved.

3. The principal causes requiring amputation of the arm are: first, injuries, where the part of the limb below is pulseless and dead; secondly, where gangrene from disease has destroyed the limb; thirdly, where cancer of the member is so situated as to be incapable of full extirpation without amputation.

Severe compound and comminuted fractures of the shaft of the humerus and even of the elbow or the shoulder, do not require amputation, if there is circulation in the part below. Extensive laceration of the soft parts with comminution of the bone makes no difference. Modern surgeons are not appalled by the ghastly looks of the wound. The bone and all the skin and muscles may be severed, but if the artery, and some of the nerves and veins are left, the limb may usually be saved.

In like manner, no one now thinks of amputating the arm for caries of the joints, nor for necrosis of the shaft of the humerus, unless some special circumstances render it necessary to disregard the usual principles.

In short, the conservative surgeons have largely won the day, so far as the superior extremity is concerned. At the same time the amputations, if required, are far less dangerous than those of the lower extremity. The primary operations are a little less fatal than the secondary, and the pathological ones (amputations of complaisance excepted) are less than half as dangerous as the traumatic. The mortality of all amputations of the arm in the Lake States is, taking all kinds together, only 11 per cent., which is about one-third the mortality abroad.

Demme, Stromeyer, and Max Schmidt (Schmidt's Jahrbücher, 1872,) agree that in gunshot wounds of the elbow joint, conservative treatment is four times more dangerous than resection, while amputation of the arm is intermediate between them. They recommend the conservative treatment, therefore, for mild cases only, and amputation only for cases not admitting of resection. Legouest, (Traité de Chirurgie d' Armée, p. 530,) says, speaking of military surgery, "When the elbow has received a comminuted fracture, and the brachial artery is opened, it is necessary to amputate the arm immediately." In my opinion this should depend on whether the collateral circulation keeps up the supply of blood. If it does, and if some of the large nerves are also intact, resection should be preferred.

### AMPUTATIONS AT THE ELBOW JOINT.

Of these I find only two Lake State cases, both of which recovered.

### TABLE V.

AMPUTATION AT THE ELBOW JOINT.

| OPERATOR. | REASON FOR OPERATION. | Complications | Time. | Result. | Practice. |
|---|---|---|---|---|---|
| Dr. E. Andrews | Mortification forearm after wound | None ... | Second'y | Recover. | Priv. pr. |
| Cook Co. Hosp. opr. not stated | Not stated | Not stat. | Primary . | " | Hosp... |

No conclusions can be drawn from so small a number. The operation abroad seems equally rare, so that the entire literature of surgery does not furnish us the means of comparing primary, secondary and pathological cases. I find only the following records:

| AUTHORITIES. | CASES. | DEATHS. |
|---|---|---|
| Pennsylvania Hospital | 1 | 0 |
| Dr. Herrgalt, Siege of Strasburg, 1870–71 | 2 | 2 |
| Other reports in Franco-German War, Deutsch. Zeit. für Chirurgie, B. II, S. 105 | 11 | 6 |
| Circular No. 6, Surg. Gen. U. S. Army | 19 | 0 |
| Statist. des Hôp. de Paris, 1861-2-3 | 4 | 2 |
| Guy's Hosp. Reports | 1 | 0 |
| Leeds' Gen. Infirmary, Mr. Nunneley | 20 | 1 |
| Zurich Hosp., 1860–67, Arch. Klin. Chir., Bd. X., S. 891 | 2 | 1 |
| Deutsch. Zeit. für Chir., Bd. II., S. 380 | 1 | 1 |
| Totals | 61 | 13 |

Mortality, 21 per cent.

This gives fifteen per cent. better results than amputation of the arm, so that it would seem it should be preferred to the latter whenever the choice of location is offered.

### OPINIONS OF AUTHORS AND CONCLUSIONS.

Expectant treatment of gunshot wounds of the elbow is to be advised only in the slightest cases. The statistics of the Med. and Surg. History of the War of the Rebellion, p. 829, part II, Surg. Vol., give 938 cases treated conservatively, with only 10 per cent. of deaths; but a great number of these were trivial wounds, and not at all to be classed with those where a bullet had gone through the interior of the joint. I think that in the latter class expectant treatment would be the most dangerous of all procedures.

Amputation at the elbow was first done by Paré, and improved by Brasdor. It gave rise to great differences of opinion among eminent men.

Those opposing or discouraging it are Boyer, Richerand, J. Cloquet, T. J. Roux, (the latter very bitterly,) Chelius (Chelius' Surg., vol. III, p. 718), and Henry H. Smith (Smith's Surg., vol. II, p. 689).

On the other hand, we have in favor of it, Brasdor, Velpeau, Dupuytren, Malgaigne, Legouest, Hamilton, Gross, and Bryant.

Gross speaks of the operation in very high terms, both as to safety and excellence of the stump. (Gross' Syst. of Surg., vol. II, p. 1110.)

Bryant of England, also praises it in the highest terms. (Bryant's Surg., p. 953.)

It is evident that the weight of authority among living surgeons is decidedly in favor of the operation. It should not be substituted for excision, but when an amputation is inevitable, and there is room for a choice of location, the elbow is to be preferred to any point above it.

### AMPUTATION OF THE FOREARM.

Owing to the success and safety of conservative treatment of the forearm, amputations of this segment are comparatively rare. I have obtained records of only 20 cases in the Lake States, which are here subjoined :

### TABLE VI.

### AMPUTATION OF THE FOREARM.

| OPERATOR. | Age. | REASON FOR OPERATION. | COMPLICATIONS. | Operati'n | Con-diti'n | Time. | Result. | Time to d'h or r'y | Practice. |
|---|---|---|---|---|---|---|---|---|---|
| Dr. J. H. Hollister | -- | Forearm crushed by cars | Intemperate | | | Primary | Recovered | | Hosp. |
| " " " | -- | " " " " | " | | | " | " | | " |
| " " " | -- | Gunshot wound of forearm | | | Good | " | " | | " |
| " E. Andrews | -- | Injury by R. R. | None | Circular | | " | " | 2 mos. | " |
| " " " | -- | Gangrene of crushed hand | " | " | | Secondary | " | 6 weeks | Priv. pra. |
| " " " | -- | Injured by cars | | | | Primary | " | 32 days | Hosp. |
| " " " | 70 | Lupus or cancroid of hand and wrist | None | | Bad | 10 years | " | 70 " | " |
| " J. H. Hollister | 19 | Wound of forearm by saw | " | | Good | Primary | " | | Priv. pra. |
| " " " | 20 | Wrist severed by sharp instrument | " | | " | " | " | | " " |
| Cook Co. Hosp. | -- | | | | | " | " | | Hosp. |
| " " " | -- | Disease of parts | | | | | Died | | " |
| Dr. H. Wardner | 33 | Hand crushed in foundry | Considerable shock | Low. 3 | Med. | Primary | Recovered | 3½ wks. | Priv. pra. |
| " " " | 30 | " " on boat | " " | Mid. 3 | " | " | " | 3 mos. | " " |
| " E. D. Kittoe | 52 | Hand and forearm crushed in thresh. mach. | " " intem. | Low. 3 | Bad | 18 hours | " | | " " |
| " " " | 56 | Scirrhus of hand | Scirrhus of lip removed 3 | " " | " | 2 years | " | | " " |
| " E. W. Lee | 8 | Comp. fract. forearm | | Flap. | Good | Primary | " | 3 weeks | " " |
| " " " | 20 | Hand crushed | | Low. 3 | " | " | " | 4 " | " " |
| " S. S. Bedal | 45 | Comp. and com. fract. | None | Cir. low. 3 | " | " | " | | " " |
| " S. Marks | 28 | Hand crushed | | Lower 3d | " | Secondary | " | 3 weeks | Hosp. |
| " " " | 50 | Erysipelas and gangrene | | Upper 3d | Bad | 4 weeks | Died | 12 days | Priv. pra. |

RECAPITULATION.

| | CASES. | DEATHS. | PER CT. MORT. |
|---|---|---|---|
| Total number | 20 | 2 | 10 |
| Traumatic Primary | 14 | 0 | 0 |
| " Secondary | 2 | 0 | 0 |
| Pathological | 4 | 2 | 50 |
| Hospital Cases | 9 | 1 | 11 |
| Private Practice | 11 | 1 | 9 |

AMPUTATION OF THE FOREARM ABROAD.

The following are the principal published records of this operation:

TRAUMATIC PRIMARY.

| AUTHORITIES. | CASES. | DEATHS. |
|---|---|---|
| Med. & Surg. Hist. War of Rebel., Surg. Vol., Part II., p. 967 | 1,0 07 | 97 |
| New York Hosp., reported by Sir J. Y. Simpson | 10 | 2 |
| Pennsylvania Hosp., " " " " | 83 | 5 |
| Boston City Hosp., Dr. Cheever | 9 | 0 |
| Mass. Gen. Hosp., reported by Sir J. Y. Simpson | 29 | 7 |
| Guy's Hosp., London | 16 | 1 |
| St. Bartholomew's Hosp., London, 1853 to 1871 | 48 | 2 |
| St. George's " " | 1 | 0 |
| U. S. Marine Hosp. | 1 | 0 |
| Dr. Herrgalt, Strasburg, French and German War | 4 | 0 |
| Dr. Beck, Austrian and Prussian War | 3 | 0 |
| Leeds Gen. Infirmary, Mr. Nunneley | 60 | 4 |
| Siege of Antwerp, Schmidt's Jahrbücher, B. 136 | 6 | 1 |
| Crimean War, " " " | 175 | 35 |
| German-French War, " " " | 10 | 2 |
| Dr. E. Warren's Surg., p. 396, Confederate Army | 23 | 2 |
| British Mil. Hosp. in Brussels, 1815, Guthrie | 22 | 1 |
| Totals, | 1,507 | 159 |

Mortality, 11 per cent.

TRAUMATIC INTERMEDIARY.

| AUTHORITIES. | CASES. | DEATHS. |
|---|---|---|
| Med. & Surg. Hist. War of Rebel., Surg. Vol., Part II., p. 967, | 450 | 106 |

Mortality, 23 per cent.

INTERMEDIARY AND SECONDARY CASES COMBINED.

| AUTHORITIES. | CASES. | DEATHS.* |
|---|---|---|
| Boston City Hosp., Dr. Cheever | 2 | 0 |
| New York " Works of Sir J. Y. Simpson | 3 | 1 |
| Pennsylvania " " " " " | 11 | 4 |
| Mass. Gen. " " " " " | 12 | 2 |
| Guy's " London | 1 | 0 |
| St. Thomas' " " | 1 | 0 |
| St. Bartholomew's Hosp., London, 1853—71 | 14 | 2 |
| St. George's " " | 1 | 0 |
| Chinese Missionary " | 1 | 0 |
| Geissel in French and German War | 5 | 0 |
| Beck in Austrian and Prussian War | 5 | 1 |
| Crimean War, Schmidt's Jahrbücher, B. 136 | 96 | 56 |
| German-French War, " " " | 2 | 2 |
| Dr. E. Warren's Surgery, p. 396, Confederate Army | 22 | 4 |
| British Mil. Hosp. in Brussels, 1815, Guthrie | 17 | 5 |
| Totals, | 193 | 77 |

Mortality, 40 per cent.

PURELY SECONDARY (AFTER INTERMEDIARY PERIOD).

| AUTHORITY. | CASES. | DEATHS. |
|---|---|---|
| Med. & Surg. Hist. War of Rebel., Surg. Vol., Part II, p. 967, | 184 | 29 |

Mortality, 16 per cent.

PATHOLOGICAL CASES.

| AUTHORITIES. | CASES. | DEATHS. |
|---|---|---|
| Boston City Hospital, Statement of Sir. J. Y. Simpson | 6 | 2 |
| New York Hospital, " " " | 7 | 2 |
| Pennsylvania " " " " | 6 | 0 |
| Mass. Gen. " " " " | 27 | 4 |
| Edinburg Infirmary, 1859—68 | 7 | 3 |
| Glasgow " 1847—68 | 23 | 6 |
| St. George's Hospital, London, 1864—68 | 8 | 4 |
| Guy's " " 1861—68 | 13 | 5 |
| London " " 1862—68 | 5 | 0 |
| Middlesex " " 1867—68 | 1 | 1 |
| King's College " " 1863—68 | 1 | 1 |
| Royal Free " " 1862—68 | 1 | 1 |
| Westmininster " " | 4 | 1 |
| St. Thomas " " | 2 | 0 |
| St. Bartholmew's" " 1853—71 | 18 | 1 |
| Leeds Gen. Infirmary, Statem't of Mr. Nunneley | 21 | 3 |
| Billroth's Practice | 4 | 2 |
| Other European Cases | 8 | 0 |
| Totals | 162 | 36 |

Mortality, 22 per cent.

GENERAL SUMMARY OF AMPUTATION OF THE FOREARM ABROAD.

| | CASES. | DEATHS. | PER CENT. MORT. |
|---|---|---|---|
| Traumatic, primary | 1,507 | 159 | 11 |
| " intermediary | 450 | 106 | 23 |
| Intermediary and secondary combined | 193 | 77 | 40 |
| Purely secondary | 184 | 29 | 16 |
| Pathological | 162 | 36 | 22 |
| Totals | 2,496 | 407 | 16 |

Mortality in the Lake States, 10 per cent.

It appears, therefore, that the mortality of this operation among us is less than two-thirds that of the published statistics.

OPINIONS OF AUTHORS AND CONCLUSIONS.

Authors have very little to say on the indications for amputation of the forearm, except to apply the following principles :

1. Conservative treatment is very safe.

2. The arteries and nerves pass down in several trunks, so that they are seldom all destroyed at once.

3. Artificial hands are of very little practical use.

Acting on these truths, surgeons rarely amputate the forearm, except for some injury which has already destroyed the life of the member, or some disease like cancer, which cannot be otherwise gotten rid of. In all severe compound fractures, gunshot wounds, etc., in which there is the least ground of hope that the circulation may recover itself, the effort is made to save the limb. Conservative treatment in the forearm and hand is carried to its fullest extent.

Legouest (*Chirurg. d' Armée*, p 350,) says, that when a bullet traverses the wrist in its greatest diameter, with great shattering, amputation of the forearm will be required.

AMPUTATIONS OF THE WRIST AND HAND IN THE LAKE STATES.

Of these, I find records of only eight cases, all of which recovered.

AMPUTATIONS AT THE WRIST JOINT—ABROAD.

TRAUMATIC PRIMARY.

| AUTHORITIES. | CASES. | DEATHS. |
|---|---|---|
| Med. & Surg. Hist. War of Rebel., Surg. Vol, Part II, p. 1018 | 54 | 5 |
| Pennsylvania Hospital | 8 | 0 |
| Dr. Herrgalt, in Strasburg | 2 | 0 |
| Leeds General Infirmary, Mr. Nunneley | 102 | 8 |
| Siege of Antwerp, Schmidt's Jahrbücher, p. 156 | 1 | 1 |
| Totals | 167 | 14 |

Mortality, 8 per cent.

TRAUMATIC INTERMEDIARY.

| AUTHORITY. | CASES. | DEATHS. |
|---|---|---|
| Med. & Surg. Hist. War of Rebel., Surg. Vol., Part II, p. 1018, | 7 | 1 |

Mortality, 14 per cent.

PURELY SECONDARY (AFTER INTERMEDIARY PERIOD).

| AUTHORITY. | CASES. | DEATHS. |
|---|---|---|
| Med. & Surg. Hist. War of Rebel., Surg. Vol., Part II, p. 1018, | 5 | 1 |

Mortality, 20 per cent.

TRAUMATIC, TIME NOT STATED.

| AUTHORITIES. | CASES. | DEATHS. |
|---|---|---|
| German Authors | 2 | 1 |
| U. S. Marine Hospital | 1 | 0 |
| Crimean War, Schmidt's Jahrbücher, p. 156 | 67 | 27 |
| Italian War, " " " | 13 | 6 |
| German-French War, Schmidt's " " | 8 | 0 |
| Totals | 91 | 34 |

Mortality, 37 per cent.

This increased mortality, as compared with that of the cases known to be primary, may be due to the fact that the second list is mainly made up of military cases, many of which had other injuries to determine a fatal result, yet it seems impossible to make any satisfactory solution of such palpable discrepancies.

PATHOLOGICAL CASES.

Cases, 14. Deaths, 1.

Mortality, 7 per cent.

GENERAL SUMMARY OF AMPUTATIONS OF THE WRIST.

LAKE STATES.

Eight cases. No deaths.

ABROAD.

| | CASES. | DEATHS. | PER CENT. MORT. |
|---|---|---|---|
| Primary | 167 | 14 | 8 |
| Intermediary | 7 | 1 | 14 |
| Purely secondary | 5 | 1 | 20 |
| Time not stated | 91 | 34 | 37 |
| Pathological | 14 | 1 | 7 |
| Totals, | 284 | 51 | 18 |

OPINIONS OF AUTHORS.

Legouest and Albert Malinas, in a work entitled "Conservation," etc., advise conservative treatment in gunshot fractures of the wrist, and, in support of their opinion, give the following facts, on gunshot wounds of this articulation :

| | MORTALITY OF CONSERVATIVE TREATMENT. | MORTALITY OF AMPUTATION. |
|---|---|---|
| Crimean War | 11 | 28 |
| Italian War | 18 | 25 to 46 |

Legouest says (*Chirurgie d'Armée*, p. 530) that amputation at the wrist is only required when the injury to the hand is such as to destroy the hope of any future use of it.

Joseph Lister, in Holmes' System of Surgery, vol. V., p. 655, rather discourages the operation, and thinks it no better than amputation of the forearm.

Gross, on the other hand, in his System of Surgery, vol. II., p. 1108, thinks it preferable by far to amputation of the forearm.

Erichsen says it is not often required.

Ashurst's Surgery, p. 115, says if it is done, the disarticulation should be at the radio-carpal junction.

Vidal (Pathologie Externe, Tome V., p. 646) approves the operation in suitable cases.

It appears, therefore, that authors conflict somewhat in their opinions of the operation, without any decisive scientific proof on either side. The statistics too are in hopeless contradiction. The Crimean war is said to have given a mortality of 28 per cent.; the Italian war is stated variously from 13 to 46 per cent., and the late American war at only 5 per cent. No results can be deduced from such utterly irreconcilable statements. Science must wait for a better collection of facts.

AMPUTATIONS THROUGH THE METACARPUS.

No records for the Lake States. Abroad, seventy-six cases are recorded without a death.

AMPUTATION AT THE HIP JOINT.

Of this important operation I obtain records of the following seven cases in the Lake States:

### TABLE VII.

### AMPUTATION OF THE HIP JOINT.

| OPERATOR. | AGE. | REASON OF THE OPERATION. | COMPLICATIONS. | Operation. | Condition. | Time. | Result. | Time to d'h | Practice. | Causes of death—remarks. |
|---|---|---|---|---|---|---|---|---|---|---|
| E. Powell | .. | Caries of hip and inflamation of knee | Pthisis pulmonalis | Flap | Bad | | Recove. | | Hosp. | Died 10 m. after of phthisis |
| " " | 45 | Recurrence of cancer in stump of thigh | | | Med. | | " | | | |
| " " | 17 | Comp. and commin. fract. entire femur | Great shock | | " | 12 hours | " | | Hosp. | |
| Ammerman | 16 | Necrosis of entire femur | Emaciation, hectic | Flap | Bad | 3 mos. | Died | | " | Exhaustion |
| D. Brainard | .. | Enchondroma of femur | | | | | Recove. | | | |
| " " | .. | Recurrence cancer removed from thigh | | | | | Died | 4 days | | Exhaustion |
| M. Waterhouse Sauk Co. Wis. | 40 | Both thighs crushed by R. R. | None | Hip u. 3 & op. th | | Primary | " | 12 'h. | Priv.p. | Shock |

RECAPITULATION.

| | | | |
|---|---|---|---|
| Total number of cases | 7 | Died | 3 |
| Traumatic | 2 | " | 1 |
| Pathological | 5 | " | 2 |

AMPUTATION OF THE HIP JOINT ABROAD.

The Surgeon-General of the U. S. Army, in Circular No. 2, publishes a report of Asst. Surg. Geo. A. Otis, M. D., which carefully collects the published cases of the world up to 1869 (Cir. No. 2, S. G. O.), so far as performed for gunshot wounds. Of these, 115 were in the Crimean and other foreign wars; 62 were in the American war, and 6 were later cases. Asst. Surg. Otis gives in Circular No. 2, p. 112, the following condensation of the whole:

AMPUTATIONS AT THE HIP JOINT FOR GUNSHOT WOUNDS ABROAD.

| | CASES. | DIED. | PER CENT. MORTALITY. |
|---|---|---|---|
| Primary (finished cases) | 76 | 75 | 99 |
| Intermediary | 76 | 70 | 92 |
| Secondary (after intermediary) | 20 | 13 | 65 |
| Re-amputations | 8 | 4 | 50 |
| Totals | 180 | 162 | 90 |

The New York, Boston and Mass. general hospitals give five traumatic primary cases, all fatal.

AMPUTATION AT THE HIP JOINT FOR PATHOLOGICAL CAUSES.

| AUTHORITIES. | CASES. | DIED. |
|---|---|---|
| Lake State Surgeons (see table No. VII. above) | 5 | 2 |
| Guy's Hosp. Reports | 1 | 1 |
| St. Thomas' Hosp. Reports | 2 | 0 |
| St. Bartholomew's Hosp., 1853 to 1863, Mr. Callender | 1 | 0 |
| Statist des Hôpit. de Paris, 1861–2–3 | 3 | 3 |
| Mass. Gen Hosp. | 2 | 2 |
| Leed's Gen. Infirmary, Mr. Nunneley | 2 | 1 |
| Ashurst's Surgery, p. 131 | 42 | 18 |
| Totals | 58 | 27 |

Mortality in pathological cases, 47 per cent.

OPINIONS OF AUTHORS AND CONCLUSIONS.

Opinions on this amputation have formerly been widely conflicting, but as statistics have accumulated and thrown light on its results, a greater degree of unanimity has been attained.

In 1740, the Academy of Surgery in Paris opposed the operation, when one of its members wished to perform it. In 1848 they approved it. In 1859 they again discussed it, and of forty-four opinions, thirty-four justified it. (*Pathologie Externe par Vidal, Tome V., p.* 703.)

Chelius, vol. III., p. 689, justifies it when a crushing or mortification extends so high as to prohibit amputation below the trochanter.

Stromeyer (*Maximen der Kriegsheilkunst*) declared in 1861 that it was not yet proved justifiable in military surgery.

Loeffler (*Grundsätze und Regehr für die Behandlung der Schusswunden im Kriege*) took similar ground. Rochard, in Saurel's *Traité de Chirurgie Navale*, pronounced it improper in the primary stage, and Sedillot maintained for years that the primary amputation was never successful.

Baron H. Larrey and M. Legouest (*Memoires de la Societé de Chirurgie, Tome V.*) obtained a definite opinion from the Society of Surgery, that the operation was unjustifiable unless the thigh was almost torn away from the trunk.

Erichsen's Surgery, vol. II., p. 301, seems to speak rather flippantly and without consideration of the terrible danger of the operation. It advocates it, not only where the disease of the femur is too extensive for excision, but even for limbs rendered simply useless by atrophy, deformity, etc. Curiously enough, in disregard of the fact that amputation is more dangerous than excision, the author recommends it as a choice, where the health is supposed to be too low to bear the excision.

Joseph Lister, in Holmes' System of Surgery, vol. V., p. 651, says it is justifiable in some desperate circumstances.

Henry H. Smith (Prin. and Pract. of Surg., vol. II., p. 694,) assumes that it may be required, and forbids the circular operation as specially objectionable.

Gross (System of Surgery, vol. II., p. 1127,) says, amputation at the hip is never to be undertaken except where there is no other chance of life.

Asst. Surg. Geo. A. Otis, M. D., the author of Circular No. 2, S. G. O., after a careful survey of the war records, and of European opinions and military experience, arrives for gunshot cases at the following conclusions (Cir. No. 2, pp. 122, 123):

"Amputation at the hip joint, for gunshot injury, notwithstanding its great fatality, cannot be altogether discarded, and should be performed under the following circumstances: 1. When the thigh is torn off, or the upper extremity of the femur comminuted with great laceration of the soft parts, in such proximity to the trunk that amputation in the contenuity is impracticable. 2. When a fracture of the head, neck or trochanter of the femur is complicated with wound of the femoral vessels. 3. When a gunshot fracture, involving the hip joint, is complicated by a severe compound fracture of the limb lower down, or by a wound of the knee joint.

"There are two other possible contingencies under which primary or early intermediate coxofemoral amputations for injury may be admissible: 1. When, without fracture, a ball divides the femoral artery and vein near the crural arch. 2. When a gunshot fracture in the trochanteric region is complicated by such extensive longitudinal fissuring as to preclude excision. Experience has yet determined nothing on these points. Secondary amputations and re-amputations at the hip, in military surgery, should be performed when, from caries, or necrosis, or chronic osteomyelitis following gunshot wounds, or amputations in the continuity, the patient's life is in jeopardy.

"Restricted to the classes of cases above enumerated, coxofemoral amputation will occasionally save lives that would otherwise be inevitably lost.

"Primary excisions of the head or upper extremity of the femur, should be performed in all uncomplicated cases of gunshot fracture of the head or neck. Intermediate excisions are indicated in similar cases where the diagnosis is not made out till late, and also in cases of gunshot fracture of the trochanters with consecutive arthritis. Secondary excisions are demanded by caries of the head of the femur, or secondary involvment of the joints, resulting from fractures in the trochanteric region or wounds of the soft parts in the immediate vicinity of the joint.

"Expectant treatment is to be condemned in all cases in which the diagnosis of direct injury to the articulation can be clearly established.

"Although the great majority of cases complicated by lesions of the pelvis terminate fatally, the successful operation of Dr. Schönborn proves that a slight injury of the margin of the acetabulum does not contra-indicate the operation of excision.

"Experience teaches that considerable portions of the shaft may be with propriety removed with the head, neck and trochanters, in cases in which splintering extends below the trochanter minor."

In the light of all the known facts, I think that these remarks of Dr. Otis are the best considered, and most carefully stated conclusions ever made, up to the time they were penned, and that to a certain extent they are applicable to other traumatic cases.

Since they were written, however, the whole system of treating wounded joints by antiseptic methods has been developed, and the question has arisen whether a certain number of shattered hips heretofore deemed to require amputation or excision would be better treated by laying open the joint freely, removing dead fragments, and treating by Lister's antiseptic methods. Probably they would, but science has not yet given the means of a positive answer, so that a painful darkness still hangs over some portions of the subject of hip joint injuries. The antiseptic consideration, however, would affect the question of excision more than that of amputation, as at the present time few would think of amputating at the hip for recent injuries, unless the limb were destroyed, or gangrene inevitable, and therefor antiseptic treatment out of the question.

There is yet one other condition in which antiseptic method might possibly come in to postpone or supersede amputation in a few rare cases. If the limb is carried away by a shot, too high for amputation below the trochanter, it has been deemed unavoidable that the patient must take the added terrible shock of a primary amputation at the hip, though his chance of surviving it is not over one in a hundred. The antiseptic method enables us in most cases to promptly subdue the local inflammation, and to completely suppress the exhausting drainage of suppuration, so that some of these cases might probably be better treated by this plan, and thus either healing the parts by granulation without operation, or else postponing the amputation to a late secondary period, when it is much safer. This principle has proved abundantly successful in some parts of the body, but there is no recorded experience of its application to hip joint wounds.

The principal pathological indication for this amputation has hitherto been cancer of the thigh, so situated as to admit of complete removal in no other way. Some instances are on record where it has been successfully done and the patient lived in comfort for years, though perhaps there may be doubt about the correctness of the diagnosis in a part of them. The whole thing lies in a nutshell. If the tumor is really malignant, there is no reasonable expectation of a permanent cure, and as pathological amputations have a mortality of 47 per cent., they have about one chance in two of killing the patient at once. The question therefore is this: Granted that there is no rational expectation of a permanent cure, and only a moderate hope of prolonging the life, is an operation, which kills immediately one-half the patients, desirable? It is evident that the prospect is greater for shortening than for lengthening the life by the operation, in such circumstances. It would seem to be justified, therefore, only in those cases where the terrible pains of the disease call for operative relief, at almost any risk.

Caries extending far down the femur, can hardly be called an indication for amputation at the hip, now that we understand the value of subperiosteal excision of bony shafts.

(To be Continued.)

